# Upconversion of Light into Bright Intravalley Excitons via Dark Intervalley Excitons in hBN-Encapsulated WSe_2_ Monolayers

**DOI:** 10.1021/acsnano.1c08286

**Published:** 2021-11-04

**Authors:** Joanna Jadczak, Mikhail Glazov, Joanna Kutrowska-Girzycka, Janina J. Schindler, Joerg Debus, Ching-Hwa Ho, Kenji Watanabe, Takashi Taniguchi, Manfred Bayer, Leszek Bryja

**Affiliations:** † Department of Experimental Physics, Wrocław University of Science and Technology, Wybrzeże Wyspiańskiego 27, 50-370 Wrocław, Poland; ‡ Ioffe Institute, 194021 St. Petersburg, Russia; ¶ Experimental Physics 2, TU Dortmund University, 44227 Dortmund, Germany; § Graduate Institute of Applied Science and Technology, National Taiwan University of Science and Technology, Taipei 106, Taiwan; ∥ National Institute for Materials Science, Tsukuba, Ibaraki 305-0044, Japan

**Keywords:** inter- and intravalley excitons, electron−phonon interaction, electron−electron interaction, upconversion, photoluminescence, WSe_2_ monolayer

## Abstract

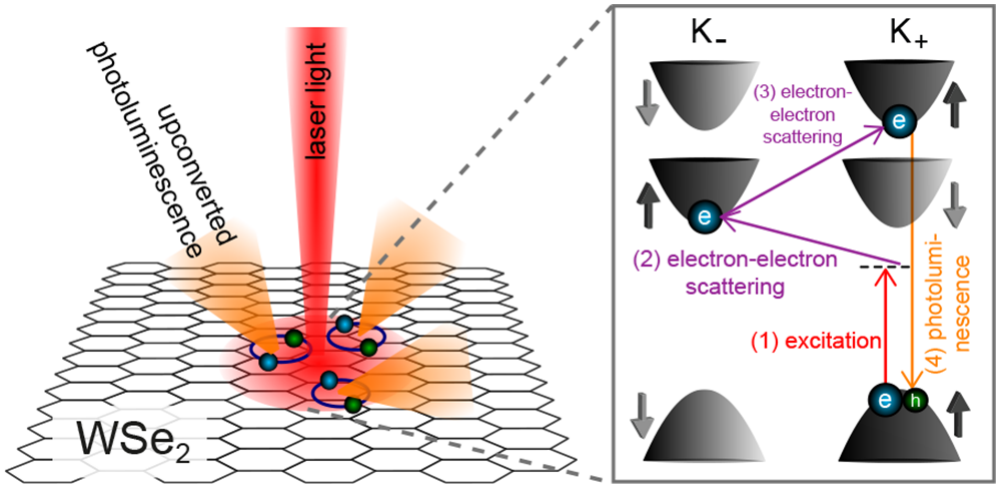

Semiconducting monolayers of transition-metal dichalcogenides are outstanding platforms to study both electronic and phononic interactions as well as intra- and intervalley excitons and trions. These excitonic complexes are optically either active (bright) or inactive (dark) due to selection rules from spin or momentum conservation. Exploring ways of brightening dark excitons and trions has strongly been pursued in semiconductor physics. Here, we report on a mechanism in which a dark intervalley exciton upconverts light into a bright intravalley exciton in hBN-encapsulated WSe_2_ monolayers. Excitation spectra of upconverted photoluminescence reveals resonances at energies 34.5 and 46.0 meV below the neutral exciton in the nominal WSe_2_ transparency range. The required energy gains are theoretically explained by cooling of resident electrons or by exciton scattering with Λ- or *K*-valley phonons. Accordingly, an elevated temperature and a moderate concentration of resident electrons are necessary for observing the upconversion resonances. The interaction process observed between the inter- and intravalley excitons elucidates the importance of dark excitons for the optics of two-dimensional materials.

## Introduction

**Figure 1:**
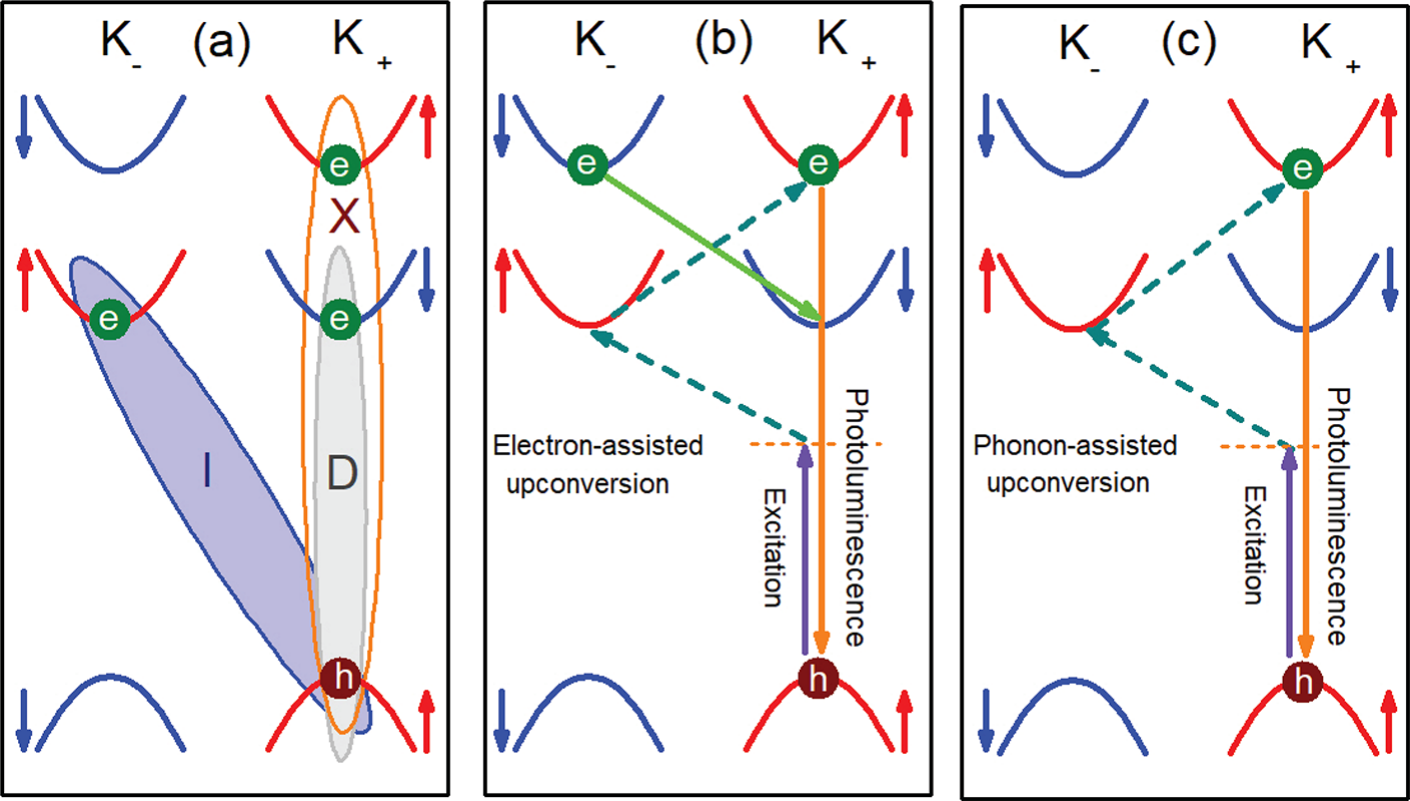
(a) Band configurations for the bright intravalley exciton (X), dark spin-forbidden intravalley exciton (D), and dark momentum-forbidden intervalley exciton (I). (b) Illustration of the upconversion assisted by intervalley scattering of a resident electron, see text and Supporting Information for details. (c) Upconversion process from the momentum-forbidden intervalley exciton to the bright intravalley exciton mediated by chiral phonons.

Layered van-der-Waals heterostructures based on transition-metal dichalcogenide (TMDC) monolayers attract attention due to prominent interaction effects which are determined by strong exciton–phonon and exciton–electron coupling. The two-dimensional confinement of charge carriers in TMDC monolayers leads to a reduced dielectric screening and to pronounced many-body effects mediated by the Coulomb interaction.^1−6^ The optical properties of TMDC monolayers are governed by strongly bound excitons^7,8^ and by various higher-order excitonic complexes whose binding energies considerably exceed those observed in conventional low-dimensional semiconductor structures.^9,10^ Additionally, owing to the strong spin–orbit coupling in TMDC monolayers and the resulting valley contrasting spin-splitting of the energy gap at the *K*_–_/*K*_+_-points, the excitonic complexes possess both spin and valley degrees of freedom.^11^ Thus, a large number of excitonic features positioned energetically below the bright intravalley exciton has been observed in the low-temperature emission spectra of tungsten-based materials. The inverted order of the optically allowed and optically forbidden states in the *K*_–_/*K*_+_-valley results in a dark exciton band lying at lower energy than the bright band;^12^ see also Figure 1(a). The involved transitions have been identified as bright “singlet” and “triplet” trions,^13,14^ neutral and charged bright biexcitons,^15,16^ spin-forbidden dark excitons^17,18^ (denoted by D in Figure 1(a)), dark (gray) trions^19,20^ and momentum-indirect dark excitons activated by scattering with defects or phonons,^1,21^ denoted by I.

Here, we exploit an alternative route to address the different excitonic species in TMDC monolayers: We use upconversion (UPC) photoluminescence (PL) where, in contrast to conventional PL measurements, emission is detected at energies above the excitation energy.^3,5,22−25^ The process of the UPC PL is thus accompanied by an energy gain. The required energy is taken from quasiparticles in the monolayer; thus, the UPC PL provides information on both the energy spectra of the TMDC as well as the scattering related to exciton–exciton, exciton–electron, and exciton–phonon interaction.^5,22,23^ We perform UPC PL measurements in hBN/WSe_2_/hBN/SiO_2_/Si heterostructures with variable thickness of the bottom hBN layer, resulting in different two-dimensional electron gas (2DEG) concentrations. The sample is excited below the bright intravalley exciton energy in the nominal transparency window of the TMDC and the emission is collected from the bright exciton with an energy gain up to ∼50 meV. From studying the emission intensity as a function of the excitation energy we reveal resonances in the upconversion photoluminescence excitation (UPC PLE) spectra. In addition to the expected resonances due to the Coulomb-bound complexes, trions and biexcitons, we observe two additional lines 34.5 and 46.0 meV below the bright exciton. These lines are most pronounced in structures whose electron concentration is estimated to (1–2) × 10^11^ cm^–2^ at temperatures ranging between 60 and 80 K. Based on their energies, these lines can be associated with UPC processes involving indirect–intervalley–excitons whose precise energy positions and optical manifestations are currently strongly debated.^1,21,26^

The emergence of indirect excitons in UPC is highly unusual and calls for an investigation of the underlying mechanisms. We propose an upconversion mechanism mediated by the dark intervalley exciton in the presence of resident electrons. The required energy and momentum are provided by the photogenerated and resident electrons which scatter between the upper and lower spin subbands in the *K*_–_- and *K*_+_-valleys; see Figure 1(b). The UPC is accompanied by the cooling of the resident charge carriers and, depending on the relation between the conduction-band spin splitting and the exchange splitting of the exciton, the UPC may be enhanced due to resonances in the intermediate state. Corresponding calculations predict a shape of the UPC PLE spectrum in reasonable agreement with the experiment. We also discuss another intrinsic mechanism of the UPC PL related to exciton–phonon interaction, Figure 1(c), in which chiral intervalley phonons provide the energy and momentum. The possible role of defects in the UPC mediated by the dark intervalley exciton is also addressed. The demonstrated UPC effect involving optically inactive exciton states may help to clarify debated uncertainties in the interpretation of intervalley exciton spectra.

## Results and Discussion

### Photoluminescence of Bright and Dark Excitons and Trions

WSe_2_ monolayers encapsulated in hBN were studied with different thicknesses of the hBN bottom-layer varying from 10 to 250 nm. The hBN top-layer is about 10 nm for all heterostructures. An additional hBN layer placed between the TMDC monolayer and SiO_2_/Si substrate acts as a buffer layer and affects the doping level in the monolayer.^5,27,28^ This impact is likely due to charged defects or inhomogeneities in the charge distribution at the SiO_2_ surface.^29^ The electron density in the semiconducting monolayer is moreover influenced by, for example, photodoping effects^30^ or intrinsic and rotationally created defects.^31,32^ The latter behave as acceptors and lead to a significant *p*-doping in the WSe_2_ monolayer.^31^ In monolayers which are embraced by hBN layers, the photodoping effect caused by laser excitation is reduced. Hence, changing the thickness of the hBN bottom-layer tunes the electron concentration in the WSe_2_ monolayer.

**Figure 2:**
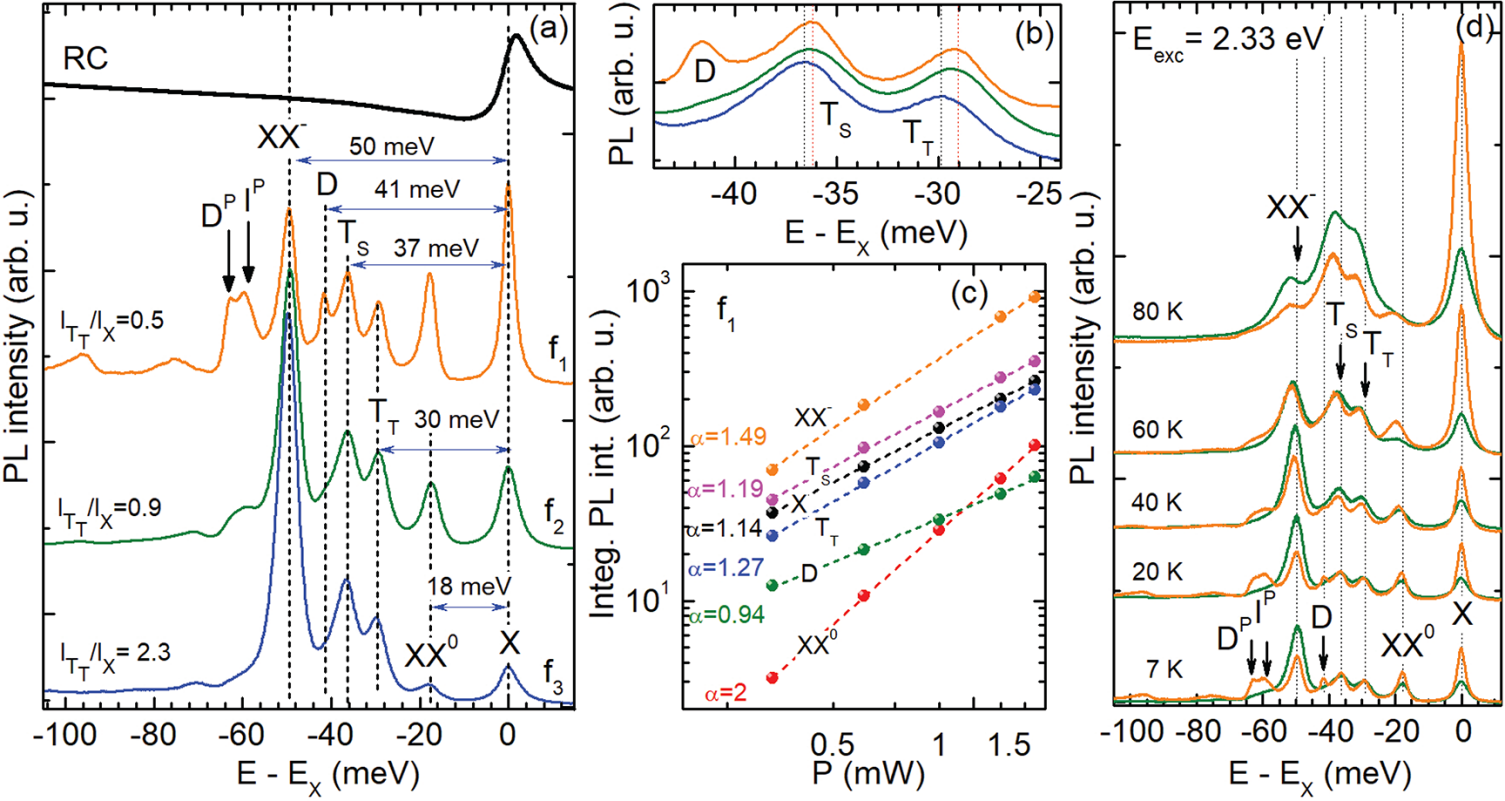
(a) PL spectra of hBN/WSe_2_/hBN heterostructures with varying thickness *d* of the hBN bottom-layer measured at *T* = 7 K: *d* = 240 nm (*f*_1_, orange), 30 nm (*f*_2_, green), 14 nm (*f*_3_, blue). The curves are vertically offset for clarity. A reflectance (RC) spectrum with the neutral exciton resonance is shown for comparison. (b) PL spectra of trions in the *f*_1_, *f*_2_, and *f*_3_ heterostructures. (c) Integrated PL intensities of the excitonic complexes X, XX^0^, T_T_, T_S_, X_G_, and XX^–^ in dependence on the laser power, exemplarily shown for sample *f*_1_. (d) PL spectra as a function of temperature ranging from 7 to 80 K, for samples *f*_1_ (orange) and *f*_2_ (green). The laser power was *P* = 0.3 mW.

Figure 2(a) compares the low-temperature PL spectra of three hBN-encapsulated WSe_2_ monolayers with different thicknesses *d* of the hBN bottom-layer: flake f_1_ with *d* = 240 nm (orange curve), *f*_2_ with *d* = 30 nm (green curve), and *f*_3_ with *d* = 14 nm (blue curve). The PL spectra are excited nonresonantly at an energy of 2.33 eV. For all structures, we observe prominent PL lines which were already reported in several previous works on WSe_2_ monolayers. Accordingly, we attribute the energetically highest peak to the neutral exciton (X). Since its energy position slightly changes from flake to flake between 1.719 and 1.732 eV, for the energy scale in each spectrum the neutral exciton energy is chosen as reference so that the energy difference *E*–*E*_X_ is shown. The emission peak, positioned 18 meV below X, is attributed to the neutral biexciton XX^0^.^15,16^ The two transitions observed about 30 and 37 meV below X are assigned to the spin-triplet and spin-singlet trions (T_T_ and T_S_), respectively.^33,34^ The feature at *E*–*E*_X_ ≈ –50 meV may be identified as charged biexciton (XX^–^).^15,16^

As clearly seen, the PL lines of these bright excitonic complexes differ in their intensities. To estimate qualitatively the electron doping in the hBN/WSe_2_/hBN structures we compare the PL intensities of the charged triplet trion (*I*_T_T__) and of the neutral exciton (*I*_X_),^4,5,35,36^ that is, we calculate the ratio *I*_T_T__/*I*_X_. It takes values of 0.5, 0.9, and 2.3 for the flakes *f*_1_, *f*_2_, and *f*_3_, respectively. This indicates an electron concentration growing with decreasing hBN bottom-layer thickness. This trend is observed for all heterostructures studied and it is consistent with the slightly increasing exciton–trion energy splitting in the samples *f*_1_, *f*_2_, and *f*_3_, respectively. As seen in Figure 2(b), the two trions T_T_ and T_S_ in sample *f*_1_ are red-shifted with respect to the neutral exciton X by 29 and 36 meV, correspondingly. In sample *f*_3_ these values increase by 1 and 0.5 meV, respectively. From the energy difference between the neutral exciton X and trions we evaluate the Fermi (F) level using the formula Δ*E* = *E*_X_–*E*_T_ = *E*_b_ + *E*_F_, where *E*_b_ corresponds to the trion binding energy. The binding energies of the T_T_ and T_S_ trions determined in a gated hBN-encapsulated WSe_2_ monolayer are equal to 28.6 and 35.4 meV, respectively.^37^ Accordingly, the Fermi level evaluated from the energy splitting between the X and T_T_ lines in the PL spectra of the structures *f*_1_ and *f*_3_ ranges from 0.4 to 1.4 meV, correspondingly, while the Fermi level followed from the X-T_S_ energy splitting ranges from 0.6 to 1.1 meV. Considering the equation *n* = *m*_e_*E*_F_/πℏ^2^ and the electron effective mass *m*_e_ = 0.4*m*_0_,^38^ we estimate the intrinsic 2D electron concentration in our structures: It lies in the range of (0.7–2.3) × 10^11^ cm^–2^ and (1.0–1.8) × 10^11^ cm^–2^.

In the PL spectrum of flake *f*_1_, which exhibits the lowest *I*_T_T__/*I*_X_ ratio and in turn the lowest electron concentration, an additional line denoted by D shows up 41 meV below the exciton PL line. This emission stems from the spin-forbidden dark exciton,^17^ which is also called a “gray” exciton.^18^ Its emission is predominantly directed along the plane of the monolayer, so that it is observed mainly due to the high numerical aperture of the microscope objective collecting the out-of-plane *p*-polarized dark exciton emission.^17^ Recent reports demonstrated that the spin-forbidden exciton in gated hBN/WSe_2_/hBN structures is observed only for low electron concentrations.^21,26,39^ This is consistent with our results, since the D line is only present in the PL spectrum of sample *f*_1_ having a lower electron concentration compared to the other two samples *f*_2_ and *f*_3_.

The well-resolved emission features *I*^P^ and *D*^P^ emerging at *E*–*E*_X_ ≈ –60 and –62 meV in the monolayer *f*_1_ are allocated to phonon-assisted recombination of the momentum- and spin-forbidden dark excitons, respectively. In particular, the *I*^P^ line arises from the coupling of the momentum–dark to the bright states via a *K*-valley phonon.^21,26^ The second peak *D*^P^ which is positioned 21.5 meV below the spin-forbidden dark exciton was interpreted as the zone-center *E*″ phonon replica of the spin–dark exciton.^1,6^ Furthermore, the low-intensity emission lines, which are observed only in the *f*_1_ monolayer between 75 and 100 meV below the X, were recently attributed to valley-phonon replicas of dark negative trions.^26^ It is worthwhile to emphasize that the observation of both the bright and dark transitions strongly depends on the electron concentration.

Complementary power- and temperature-dependent PL measurements provide a deeper insight into the nature of the excitonic complexes observed in the WSe_2_ monolayers. The laser power dependences of the intensities integrated over each PL line of sample *f*_1_ are presented in Figure 2(c). The neutral exciton X and both trions T_T_ and T_S_ exhibit an approximately linear power dependence. In accordance with a power law, *I* ∼ *P*^α^,^40^ the exponents read α_X_ = 1.14 ± 0.02, α_T_T__ = 1.27 ± 0.02, and α_T_S__ = 1.19 ± 0.02, respectively. Also, the dark exciton D reveals a linear dependence with α_D_ = 0.94 ± 0.02. The power dependence of the biexciton XX^0^ follows a quadratic behavior, whereas that of the charged biexciton XX^–^ is superlinear with α_XX^––^_ = 1.49 ± 0.03.

Let us now compare the thermal characteristics of the PL spectra for the hBN-encapsulated WSe_2_ monolayer with *d* = 30 and 240 nm, respectively. As shown in Figure 2(d), the PL intensity of the neutral exciton is enhanced by a temperature increase from 7 to 80 K. This tendency is more pronounced for the sample *f*_1_ whose exciton emission dominates the other transition lines. By comparison, for the *f*_2_ monolayer with higher electron concentration, the trion emission is more intense than that of X at high temperatures. The XX^0^ emission gradually weakens for increasing temperature in both monolayers; however, this occurs at different rates depending on the electron concentration.

Above 60 K, the XX^0^ PL, for sample *f*_2_, can hardly be distinguished from the background, while, for sample *f*_1_, it is visible even at 80 K. The spectral signatures of T_T_ and T_S_ are well resolved up to 80 K, for both samples. The PL intensity of the spin–dark exciton D observed only in *f*_1_ decreases rapidly, and above 40 K this peak is dominated by the trion line T_S_. The phonon-assisted transitions are visible as well only at low temperatures up to 40 K which results from the competition between oscillator strength and occupation probability.^1^ Since the dark exciton band is at lower energy than the bright X band, it is more likely that at low temperatures excitons recombine indirectly via a virtual state than they occupy the energetically higher bright state.^1^

### Upconversion via Intervalley Excitons

**Figure 3:**
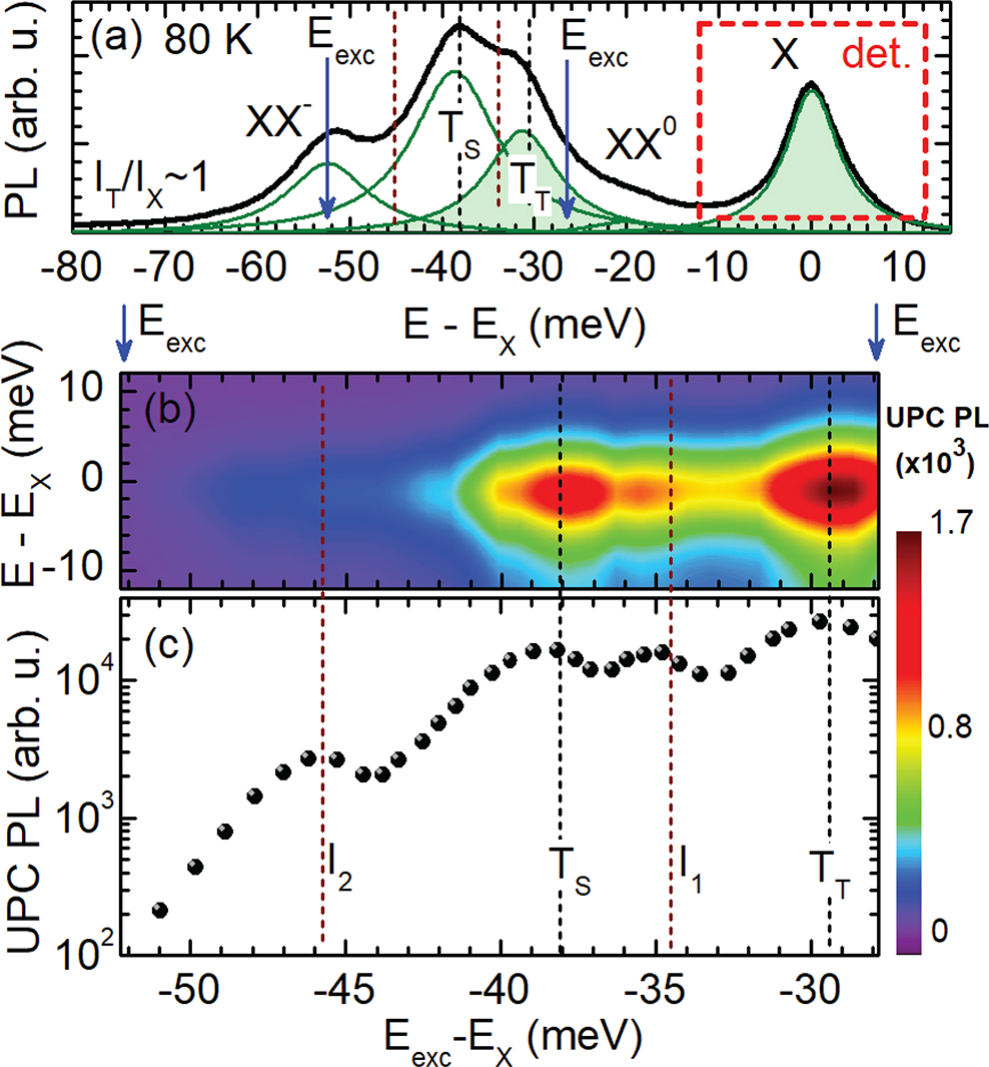
(a) PL spectrum of the hBN/WSe_2_/hBN structure (sample *f*_2_) at 80 K excited at 2.33 eV photon energy. The green lines result from a decomposition using four Lorentz functions fitted to the PL. (b) Color map of the UPC PLE spectra. (c) Integrated UPC PL of the neutral exciton for excitation energies ranging from 52 to 28 meV below the X resonance.

Figure 3 presents the first part of our key results on the excitation of excitonic upconversion PL in the hBN-encapsulated WSe_2_ monolayer (*f*_2_), measured at 80 K. In Figure 3(a) the PL spectrum of XX^0^, T_T_ and T_S_ and XX^–^ is demonstrated as a function of the energy difference of these emission lines relative to the neutral exciton X. The intensities of the triplet trion and exciton PL are similar; thus, *I*_T_T__/*I*_X_ amounts to 1. For exciting the UPC PL, we resonantly tune the laser energy between the XX^–^ peak and the high-energy flank of the T_T_ peak, marked by the blue arrows. The UPC is linked to the neutral exciton PL that is detected during scanning the laser-excitation energy, as indicated by the red dashed box in Figure 3(a). The UPC PLE spectra at 80 K are depicted in Figure 3(b) by a color map which gives the neutral exciton PL line as a function of the excitation energy *E*_exc_ detuned from *E*_X_. This energy difference (|*E*_exc_ – *E*_X_|) is denoted by the UPC energy gain *ΔE*. The integration across the UPC PL energy range yields the dependence shown in Figure 3(c). It clearly exhibits three resonances at energy gains of 29.5, 34.5, and 38.0 meV. A further weak peak is identified at about 46 meV. The resonances at 29.5 and 38.0 meV match the spectral positions of the spin-triplet and spin-singlet trion, respectively, cf. the PL spectra in Figure 2(d). The resonances at 34.5 and 46 meV, named in the following *I*_1_ and *I*_2_, respectively, correspond to transitions which were recently observed in low-temperature PL spectra in gated hBN/WSe_2_/hBN heterostructures near the neutrality point.^26^ There seems some consensus that these features may be related to dark momentum-forbidden excitons; however, their origin is still under heavy debate, see refs.^1,21,26^

**Figure 4:**
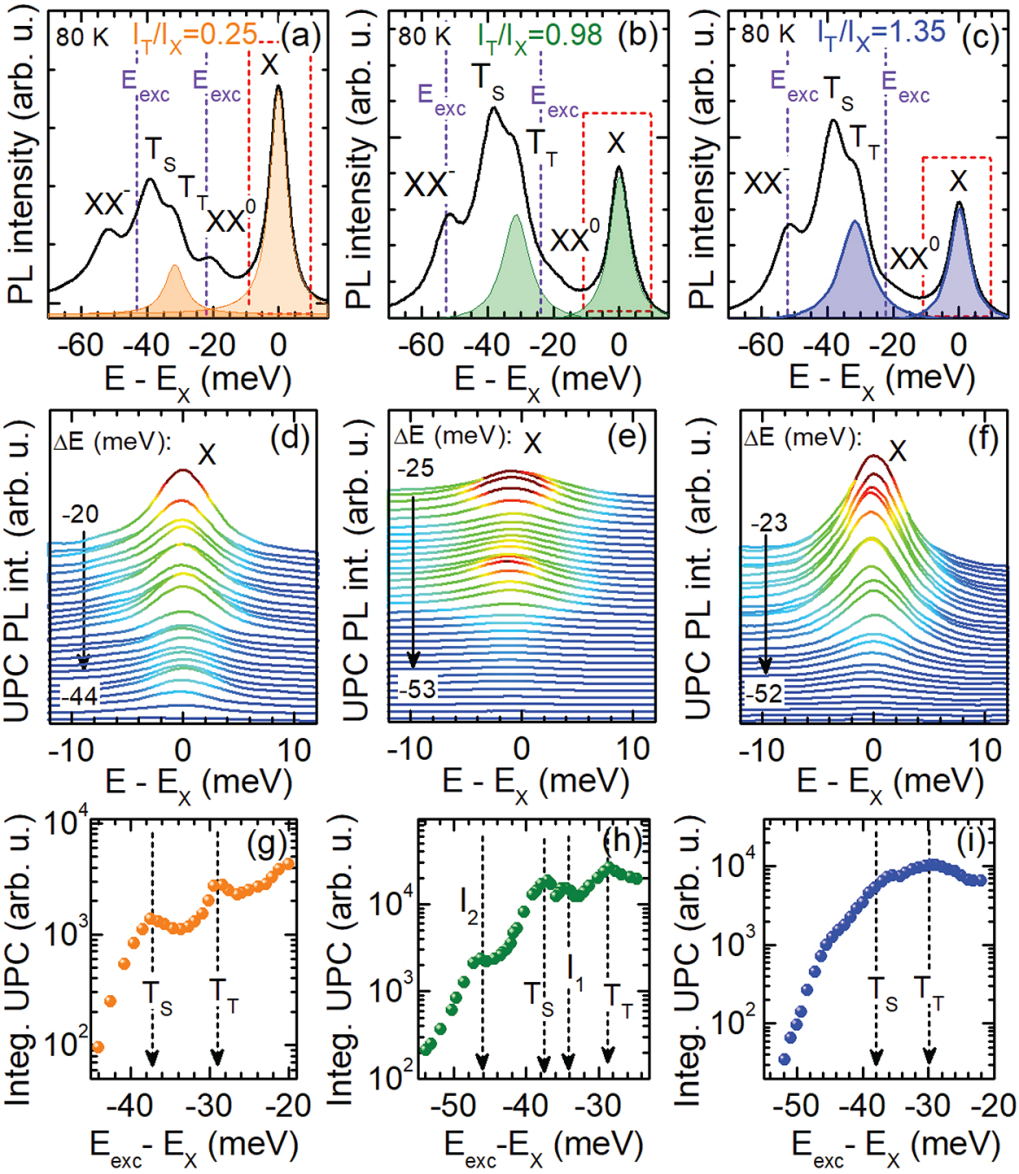
PL spectra of the hBN/WSe_2_/hBN monolayers (a) *f*_1_ [first column], (b) *f*_2_ [second column], and (c) *f*_3_ [third column] with different intensity ratios *I*_T_T__/*I*_X_; *T* = 80 K. (d–f) Examples of the UPC PL spectra of these samples for varying energy gain *ΔE*. (g–i) Integrated UPC PL of the intravalley neutral exciton as a function of *ΔE* at 80 K.

**Figure 5:**
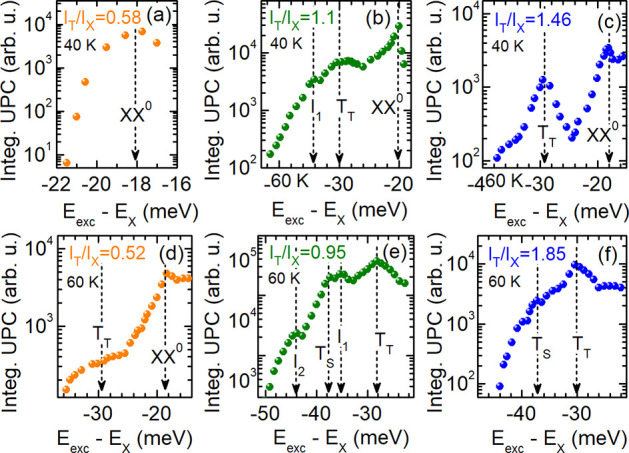
Integrated UPC PL from the intravalley neutral exciton in dependence on the energy gain *ΔE* measured at 40 K [first row] and 60 K [second row]. The data in the first [second, third] column stem from the sample *f*_1_ [*f*_2_, *f*_3_].

The dependences of the UPC PLE spectra on the temperature and electron concentration represent the second part of our experimental key results. In Figure 4 the PL spectra, the UPC PLE spectra and the integrated UPC PL measured at 80 K are shown for the three different samples having different ratios *I*_T_T__/*I*_X_; the data in the first [second, third] column belong to the sample *f*_1_ [*f*_2_, *f*_3_]. The integrated UPC PL intensities taken at 40 and 60 K are demonstrated in Figure 5. The data shown in the second column of Figure 4 coincide with that of Figure 3. For exciting the WSe_2_ monolayers resonantly between XX^–^ and XX^0^ and detecting the response of the X PL, as marked by the dashed lines in the PL spectra of panels (a), (b), and (c), we obtain UPC PLE spectra which are shown in the panels (d), (e), and (f). In these presentations, multiple resonances are identified. They are highlighted in the integrated UPC PL given in the third row of Figure 4. The UPC intensity enhancements at the T_T_ and T_S_ resonances are visible for all intensity ratios and, in turn, electron concentrations. They are, however, significantly weaker at high electron concentration; see Figure 4(i). The *I*_1_ and *I*_2_ resonances are only observed in the structure *f*_2_ for an electron density estimated to (1–2) × 10^11^ cm^–2^. This is also the case at the other temperatures of 40 and 60 K; see Figure 5.

The UPC of the spin-triplet trion T_T_ to the neutral exciton, which causes spontaneous anti-Stokes emission with an energy gain of about 30 meV, is attributed to double-resonant Raman scattering mediated by the *A*′_1_ optical phonon^3^ (see also polarization-resolved UPC PL spectra in Figure 6 in the Supporting Information). In this process, absorption of an optical phonon from the environment promotes a trion into a final state composed of an unbound electron and a neutral exciton.^3,24^ Moreover, it was recently demonstrated using Fermi’s golden rule together with the effective mass approximation that, in a WSe_2_ monolayer, both the energetically degenerate *A*′_1_ and *E*′ phonon modes contribute to the UPC process; the process strongly depends on the temperature and dielectric environment.^24^ The population transfer from the spin-singlet trion T_S_ to the neutral exciton observed in our experiment at 80 K may be explained by the same model. However, due to the higher energy gain of 38 meV the UPC is likely mediated by the absorption of two optical phonons which is consistent with the increasing probability for multiphonon absorption at elevated temperatures.^5^

**Figure 6:**
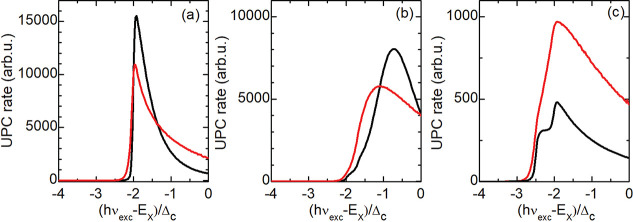
UPC rates calculated for different ratios between Δ and Δ_c_; σ = ^1^/_2_: (a) Δ = Δ_c_, (b) Δ = 2/3 Δ_c_, and (c) Δ = 3/2 Δ_c_. The black solid curves are based on eq 1 and *k*_B_*T* = Δ_c_/100, whereby the populations of the final electron states are neglected. The red dashed curves are calculated for high temperature given by *k*_B_*T* = Δ_c_/10. All resonances are artificially broadened by γ = Δ_c_/5.

In the following, we will discuss the origin of the *I*_1_ and *I*_2_ features in terms of upconverting via dark excitons to bright intravalley excitons, namely into neutral excitons X. Naturally, the excess energy required for the UPC process can be collected from either monolayer excitations (phonons, resident electrons) or from the incident laser excitation, that is, from multiphoton absorption. We observe the resonances in the UPC PLE spectra at relatively low excitation densities, so we focus on the processes related to exciton–phonon and exciton–electron coupling. The dependences of the UPC PLE spectra and, in particular, of the *I*_1_ and *I*_2_ features on the electron density moreover call for treating the UPC process by an electron-assisted mechanism which we consider first.

The electron-assisted mechanism is schematically illustrated in Figure 1(b). It consists of four steps, starting with a direct optical transition, in which an incident photon with energy ℏω_exc_ and negligible wavevector is absorbed and a virtual (direct-momentum) exciton state is formed. Due to electron–electron scattering the resident electron and the photoelectron exchange their valleys under spin conservation. Thereby, the photoelectron arrives at a real intermediate state. Subsequently, a second spin-conserving electron–electron scattering occurs, leading again to a valley switching. As a result, we obtain a bright direct exciton whose energy exceeds that of the incident photon. Finally, a photon is emitted with the energy ℏω_f_ = ℏω_exc_–Δ*E*_1_–Δ*E*_2_, where Δ*E*_*i*_ is the change in energy of a given electron (*i* = 1,2). Neglecting the Coulomb interaction in the exciton one can readily see that the process is possible when electrons at the bottom of the excited conduction subbands scatter toward the bottom of the ground spin subbands. Hence, for *k*_B_*T* ≲Δ_c_ and *E*_F_ ≲Δ_c_, where Δ_c_ is the conduction-band spin splitting, the UPC excitation occurs at *E*_X_–2Δ_c_ with an exponential tail at the low-energy side (reflecting the thermal distribution of resident electrons) and a step-like feature at ℏω_exc_ ≥ *E*_X_–2Δ_c_.

The electron-assisted UPC can be described in a way similar to the anti-Stokes Raman scattering of light, taking into account the electron–electron interaction rather than the electon-phonon interaction. It is most convenient to use the diagram technique to consider in a unified way both resonant and nonresonant contributions. In the noncrossing approximation we obtain the following expression for the UPC rate (see Supporting Information for details):
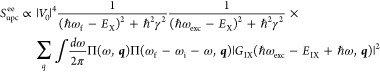
1Here, we neglect the small wavevectors of the incident and emitted photons as compared to the electron wavevector ***q*** = ***k*** – ***K*** with the transferred wavevector ***k*** and the wavevector ***K*** connecting the ***K***_+_ and ***K***_–_ valleys. *G*(ε, ***q***) = (ε – ℏ^2^*q*^2^/2*m*_IX_ + *i*γ)^−1^ is the Green’s function of the indirect momentum–dark exciton (IX), and *V*_0_ is the matrix element of the intervalley electron–electron scattering. In eq 1, Π(ω, ***q***) is the imaginary part of the electron-polarization loop related to the intervalley scattering which is given by Π(ω, ***q***) = ∑_***p***_*f̃*_***p***_(1 – *f*_***p***+***q***_) δ(ℏω – *E*_***p***_ – Δ_c_ + *E*_***p***+***q***_). Here, *E*_*p*_ = ℏ^2^*p*^2^/2*m*_e_ is the electron dispersion, *f̃*_***p***_ and *f*_***p***+***q***_ are the electron distribution functions in the upper and lower spin subbands of the conduction band. To obtain the analytical result, we assume that *k*_B_*T*, *E*_F_ ≪Δ_c_. This allows us to neglect the occupancy of the final states and also to replace ***p*** + ***q*** by ***q***. We also take into account that the upconverted PL is observed at the neutral exciton resonance ℏω_f_ ≈ *E*_X_. We further introduce the splitting between the direct and indirect excitons by Δ = *E*_X_ – *E*_IX_, the electron-to-exciton mass ratio by σ = *m*_e_/*m*_IX_, and the characteristic electron–electron scattering rate by ; the resulting simplifications are found in the Supporting Information.

The spin splitting in the conduction band was experimentally determined to be Δ_c_ = 14 meV for a WSe_2_ monolayer,^41^ while the energy difference between the inter- and intravalley excitons was measured to about Δ = 32 meV.^21^ Theoretical calculations lead to similar values.^38,42^ We thus consider the case of Δ > Δ_c_. For this case, there is no resonance in the intermediate state; a resonant electron-assisted process is established only for Δ_c_ ≥ Δ at 0 < σ < 1. The analysis of the general eq 1 in the limit of *T* →0 and γ →0 shows that, for 0 < σ < 1, two steps are expected in the UPC PLE spectra at
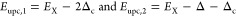
2

Figure 6 depicts the UPC rates calculated numerically using the full expression of the electron polarization operator. For Δ ≤ Δ_c_ and σ = 1/2, the spectral evolution of the UPC rate contains only one broad peak. For Δ = 3/2 Δ_c_, the excitation-energy dependent UPC rate exhibits two peaks which are attributed to the *I*_1_ and *I*_2_ features observed experimentally 34.5 and 46 meV below the neutral exciton, respectively. The peaks broaden considerably for increasing temperature. Adapting the peak positions to the experimental data, we evaluate the spin splitting of the conduction band to Δ_c_ = 17.25 meV and the energy difference between the intra- and intervalley exciton to Δ = 28.75 meV. The latter value is similar to the values reported in the refs (1 and 43)

By analogy with the recombination of momentum–dark intervalley excitons via individual phonons, we propose that the coupling between the dark intervalley and bright intravalley exciton states in the UPC process may also be mediated by single phonons and their combination, facilitating the required spin-conserving transitions. Instead of a phonon, a defect may also act as scattering partner. As sketched in Figure 1(c), after virtual exciton creation an intervalley ***K***_+_ →***K***_–_ (chiral) phonon with energy ℏΩ_*K*_ is absorbed and the electron of the exciton is transferred to the real intermediate state in the opposite valley. In the third step, the absorption of a second intervalley ***K***_–_ →***K***_+_ (chiral) phonon transfers the electron to the real final state back in the initial valley. A photon with energy ℏω_f_ = ℏω_exc_+ 2ℏΩ_*K*_ is emitted. Since ℏω_f_ is equal to the intravalley direct exciton energy *E*_X_, the peak in the UPC excitation spectra occurs at *E*_X_–2ℏΩ_*K*_. Details on the calculation of the UPC rate are provided in the Supporting Information.

The phonon-assisted mechanism for explaining the resonances *I*_1_ and *I*_2_ in the UPC process thus requires phonons with an energy ℏΩ_*K*_ of about 17.3 meV (140 cm^–1^) and 23 meV (180 cm^–1^), respectively. Helicity-resolved Raman scattering spectra were measured at 7 K, for resonantly exciting the neutral exciton at 1.753 and 1.760 eV, respectively; see Supporting Information Figure 5. The spectra reveal different phonon modes which may contribute to the exciton–phonon intervalley scattering related to the *I*_2_ feature. A possible contribution may be provided by the *K*-valley phonon mode *K*_3_ [branch LO(*E*′)^44^] with an energy of about 26 meV. It provides a dominant mechanism for an intervalley transition in the conduction band.^26^ Hence, the UPC process involving the *I*_2_ resonance may be contributed by the absorption of two chiral *K*_3_ phonons.

Finding a suitable phonon mode for the UPC feature *I*_1_ proves to be challenging. The Λ- or *K*-point LA phonons with energies of about 14 and 18 meV, respectively, may become relevant.^45^ Actually, the exciton states at the Λ-valleys play a significant role as intermediate states in exciton formation and relaxation, since the conduction band minima at these valleys are relatively close (about 35 meV) to the *K*-valley.^46,47^ The exciton–phonon scattering in the phonon-assisted UPC mechanism may occur between the *K*- and Λ-valleys, which also fits to the aforementioned theoretical model.

## Conclusions

Although the UPC mechanisms are fourth-order processes, the intensity enhancements at the *I*_1_ and *I*_2_ resonances are pronounced. They appear only in the UPC PLE spectra, while they are indistinguishable in the PL spectra due to an intrinsic electron-doping in the studied structures and, in turn, a high emission intensity of the trions and charged biexciton. The specific temperature and electron-concentration dependences of the resonances *I*_1_ and *I*_2_ in the UPC PL the intravalley exciton indicate that both the electron-assisted UPC mechanism as well as the phonon-assisted process are relevant. The energy gain in the electron-assisted UPC of the initially created virtual exciton originates from the cooling of the resident electron gas. Indeed, in the course of the spin-conserving intervalley exciton–electron interaction (second step) the resident electron loses kinetic energy, while the virtual exciton transforms to an intervalley exciton. The second exciton–electron scattering transforms the momentum–dark exciton to the bright intravalley state. Moreover, the upconversion PL has predominantly the same helicity as the exciting laser light (circular copolarization); see SI for details. This observation supports our model in which intervalley transitions of the hole are disregarded.

The spectral feature positioned at the energy of about 34.5 meV was attributed in previous studies to the momentum–dark exciton,^21,26^ where a hole and an electron are located in opposite *K*-valleys. A feature whose transition is about 46 meV below the neutral exciton was interpreted as *K*-valley phonon replica of the momentum–dark exciton.^26^ In a recent report by Brem et al.,^1^ a microscopic model predicts two different momentum–dark excitons positioned 34 and 46 meV below *E*_X_. Our experimental and theoretical data demonstrate that the resonances *I*_1_ and *I*_2_ at excitation energies detuned by –34.5 and –46 meV from the bright intravalley exciton also involve intervalley (momentum–dark) excitons; however, for the UPC, the assistance from electrons and/or phonons is additionally essential. Besides that, the observation of *I*_1_ and *I*_2_ in the narrow temperature window from 60 to 80 K and in the hBN/WSe_2_/hBN monolayers at low electron concentration ranging at about (1–2) × 10^11^ cm^–2^ is in contrast to recent reports, where the PL of momentum–dark intervalley excitons was obtained only at the neutrality points in WSe_2_ monolayers. Our results extend the current discussion on momentum–dark intervalley excitons in TMDC materials demonstrating a dark-bright-exciton UPC mechanism.

## Methods

The WSe_2_ monolayers studied here were prepared by mechanical exfoliation of bulk crystals which were grown by the chemical vapor transport (CVT) technique. Prior to the crystal growth, a powdered compound was prepared from the elements (W: 99.99%; Se: 99.999%) by chemical reaction at *T* = 1000 °C for 10 days in evacuated quartz ampules. The chemical transport was achieved with Br_2_ as transport agent having a density of about 5 mg/cm^3^. The growth temperature was set from 1030 to 980 °C with a temperature gradient of 3 °C/cm. X-ray diffraction measurements confirmed that the crystal stacking had a two-layer hexagonal (2H) structure.^48^

The hBN/WSe_2_/hBN/SiO_2_/Si heterostructures were prepared using high-purity hBN.^49^ The WSe_2_ monolayers and the hBN flakes with different thicknesses were mechanically exfoliated and then stacked using the deterministic all-dry stamping method on Si substrates (300 nm SiO_2_). To improve the contact between the transferred layers, immediately after the transfer of each subsequent layer, the sample was annealed for 20 min at a temperature of 180 °C on a hot plate in air. Additionally, after the transfer of the last hBN top-layer the heterostructure was annealed for 2 h at 200 °C in air.^50^

For the PL and PLE experiments, the samples were mounted on the coldfinger of a nonvibrating closed-cycle helium cryostat, in which the temperature could be varied from 7 to 350 K. The PL was excited by the second harmonic 532 nm (2.33 eV) of a continuous-wave single-mode Nd:YAG laser. The UPC PLE was excited by a continuous-wave Ti:sapphire laser whose emission was tunable in the range from 720 to 760 nm. The laser beam was focused on the sample under normal incidence using a high-resolution, long-working distance (WD = 10 mm, NA = 0.65) 50× microscope objective. The diameter of the excitation spot was about 1 μm. The emission from the sample was collected by the same microscope objective and was analyzed with a 0.5-m-focal length spectrometer equipped with a 600 lines/mm grating and a Peltier-cooled charged-coupled-device Si camera. The RC spectrum was measured at the same setup using a filament lamp as light source. The Raman scattering spectra were excited by the continuous-wave Ti:sapphire laser and were obtained in the backscattering geometry. To eliminate the scattered laser light a set of short- and long-pass edged filters was used.

## Supporting Information Available

Descriptions of theoretical models of the upconversion including the phonon-assisted process (Figure 1), electron-assisted process (Figure 2) with the diagrammatic representation of the electron-scattering induced upconversion (Figure 3) and the calculated upconversion rates for different model parameters (Figure 4), electron-defect processes, and the upconversion through the trion. Furthermore, complementary experimental data on the helicity-resolved Raman scattering measurements (Figure 5) and polarization-resolved upconversion PL (Figure 6) are presented. The Supporting Information is available free of charge at https://pubs.acs.org/doi/10.1021/acsnano.1c08286.
