# Supplementary material for: Upconversion of Light into Bright Intravalley Excitons via Dark Intervalley Excitons in hBN-Encapsulated WSe2 Monolayers
Source: ACS Nano. 2021 Nov 4;15(12):19165–74. doi: 10.1021/acsnano.1c08286 (PMC8717626; doi:10.1021/acsnano.1c08286)
Supplement: Supplementary file 1 — nn1c08286_si_001.pdf [file nn1c08286_si_001.pdf]

Supporting Information: Upconversion of Light into Bright Intra-Valley Excitons *via* Dark Inter-Valley Excitons in hBN-Encapsulated WSe_2 Monolayers

Joanna Jadczak,[*,†] Mikhail Glazov,[‡] Joanna Kutrowska-Girzycka,[†]
Janina J. Schindler,[¶] Joerg Debus,[¶] Ching-Hwa Ho,[§] Kenji Watanabe,[‖]
Takashi Taniguchi,[‖] Manfred Bayer,[¶] and Leszek Bryja[*,†]

†*Department of Experimental Physics, Wrocław University of Science and Technology, Wybrzeże Wyspiańskiego 27, 50-370 Wrocław, Poland*

‡*Ioffe Institute, 194021 St. Petersburg, Russia*

¶*Experimental Physics 2, TU Dortmund University, 44227 Dortmund, Germany*

§*Graduate Institute of Applied Science and Technology, National Taiwan University of Science and Technology, Taipei 106, Taiwan*

‖*National Institute for Materials Science, Tsukuba, Ibaraki, 305-0044, Japan*

E-mail: joanna.jadczak@pwr.edu.pl; leszek.bryja@pwr.edu.pl

Theoretical models of upconversion

In the upconversion process the emission is observed at higher energies as compared with the energy of the excitation[1–6]. At elevated intensities the upconversion can be caused by non-linear optical processes, including two-photon absorption, see Refs.[1,2,7,8] and references

therein for details. Energy is taken from the crystalline lattice (*via* phonon absorption)[9] or from resident electrons (*via* electron-electron scattering). This process can be considered as a anti-Stokes scattering of light. Wavevectors of the incident and emitted photons are small in comparison to the wavevectors of the electrons and phonons. The process is enhanced provided that at least some of the intermediate states are real. We consider the situation where the inter-valley exciton is generated in the intermediate state.

Phonon-assisted process

In the phonon-assisted process the energy needed for the upconversion is taken from the lattice vibrations, *i.e.*, from the phonons. Here, we consider, as an example, the situation where the inter-valley (chiral) phonon is involved and in the intermediate state the inter-valley exciton is formed.

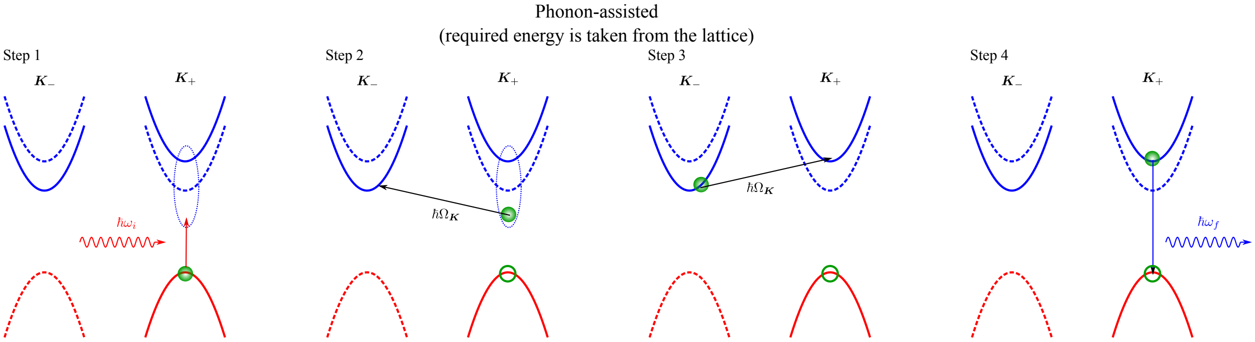

Figure 1: Schematic illustration of the phonon-assisted upconversion process. Solid parabolas show the spin-up electron dispersion and dashed parabolas show the spin-down electron dispersion. Only spin conserving transitions are considered. Excitonic effects are discarded in these schematics.

The process is schematically shown in Fig. 1 and consists of the following steps:

Step 1. Direct optical transition, incident photon $\hbar\omega_{exc}$ with the wavevector $\boldsymbol{\kappa}_{exc}$ (κ_{exc} is negligible) is absorbed and a virtual state of the direct exciton is formed, Fig. 1.

Step 2. Inter-valley $\boldsymbol{K}_+ \rightarrow \boldsymbol{K}_-$ (chiral) phonon with the energy $\hbar\Omega_{\boldsymbol{K}}$ is absorbed and the electron in the exciton is transferred to the real intermediate state in the opposite valley.

Step 3. Second inter-valley $\boldsymbol{K}_- \rightarrow \boldsymbol{K}_+$ (chiral) phonon with the energy $\hbar\Omega_{\boldsymbol{K}}$ is absorbed

and the electron in the exciton is transferred to the real final state in the opposite valley.

Step 4. The photon with the energy $\hbar\omega_f = \hbar\omega_{exc} + 2\hbar\Omega_{\boldsymbol{K}}$ is emitted. Since $\hbar\omega_f = E_X$ (intra-valley direct exciton energy), the peak in the unconversion excitation spectra occurs at

$$\hbar\omega_{exc} = E_X - 2\hbar\Omega_{\boldsymbol{K}}. \tag{1}$$

The calculation of the upconversion rate can be performed following the Ref. [10]. The result for the two-phonon process in the non-crossing approximation reads

$$S_{up}^{phonon} \propto \frac{n_o^2\Gamma}{4\Gamma^2 + (\omega_f - \omega_{exc} - 2\Omega_{\boldsymbol{K}})^2} \frac{1}{(\hbar\omega_f - E_X)^2 + \hbar^2\gamma^2} \frac{1}{(2\hbar\Omega_{\boldsymbol{K}})^2} \frac{\gamma_o}{\gamma}, \tag{2}$$

where Γ is the phonon damping rate, γ is the total exciton damping rate ($\Gamma, \gamma \ll \Omega_{\boldsymbol{K}}$), and γ_o is the exciton damping rate related to the phonon emission/absorption scattering,

$$n_o = \frac{1}{\exp\left(\frac{\hbar\Omega_{\boldsymbol{K}}}{k_B T}\right) - 1} \approx \exp\left(-\frac{\hbar\Omega_{\boldsymbol{K}}}{k_B T}\right), \tag{3}$$

is the phonon occupancy.

This model can further be extended to account for the interplay of phonon- and defect-assisted processes where the energy is taken from the phonon, while the momentum is taken from the defect. In this case, instead of Eq. (1), we obtain the peak in the upconversion rate at $\hbar\omega_{exc} = E_X - \hbar\Omega_{\boldsymbol{K}}$ and the spectrum of the upconversion takes the form similar to Eq. (2) with $2\Omega_{\boldsymbol{K}} \to \Omega_{\boldsymbol{K}}$. We also note that the energy of the bright exciton formed as a result of the upconversion may exceed E_X. Accordingly, additional relaxation of excitons towards a zero-momentum radiative state is needed. This will provide the background in the upconversion excitation spectra at $\hbar\omega_{exc} > E_X - 2\hbar\Omega_{\boldsymbol{K}}$, for the two-phonon processes, or $\hbar\omega_{exc} > E_X - \hbar\Omega_{\boldsymbol{K}}$, for the phonon+defect process.

Electron-assisted process

The alternative source of energy in the process of upconversion is the resident electron gas which is formed in doped structures. The process is schematically shown in Fig. 2 and consists of the following steps:

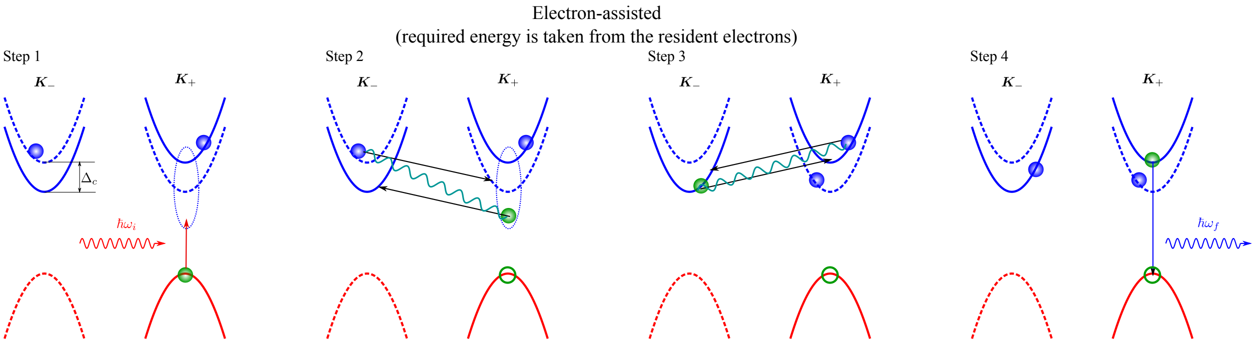

Figure 2: Schematic illustration of the electron-assisted upconversion. Solid (dashed) parabolas indicate the spin-up (spin-down) electron dispersion. Only spin-conserving transitions are considered and only electrons involved in the upconversion are shown, other resident electrons are not illustrated. Excitonic effects are discarded in these schematics.

Step 1. Direct optical transition, incident photon $\hbar\omega_{exc}$ with the wavevector $\boldsymbol{\kappa}_{exc}$ (κ_{exc} is negligible) is absorbed and a virtual state of the direct exciton is formed, Fig. 1.

Step 2. Due to the electron-electron scattering the resident electron and photo-electron scatter and change their valleys, the photo-electron goes to the real intermediate state.

Step 3. Second electron-electron scattering again leads to a valley exchange of the electrons.

Step 4. The photon is emitted with the energy $\hbar\omega_f = \hbar\omega_{exc} - \Delta E_1 - \Delta E_2$, where ΔE_i describes the change in the energy of a given electron ($i = 1, 2$). For $k_B T \lesssim \Delta_c$, where Δ_c is the conduction band spin splitting, and $E_F \lesssim \Delta_c$, the processes, in which the electrons from the bottom of the excited conduction subbands are involved, most probably realize the real intermediate state. Hence, the peak in the unconversion excitation spectrum should appear (neglecting electron-hole interaction) at

$$E_X - 2\Delta_c, \tag{4}$$

with the exponential flank at the low-energy side (reflecting the thermal distribution of the resident electrons) and a step-like feature at $\hbar\omega_{exc} \geqslant E_X - 2\Delta_c$. The inclusion of excitonic effects changes the threshold (4), see below.

The diagrammatic representation of the upconversion rate caused by the exciton-electron scattering is given in Fig. 3. One can also interpret this transition as the process involving the absorption of the inter-valley plasmon[11,12].

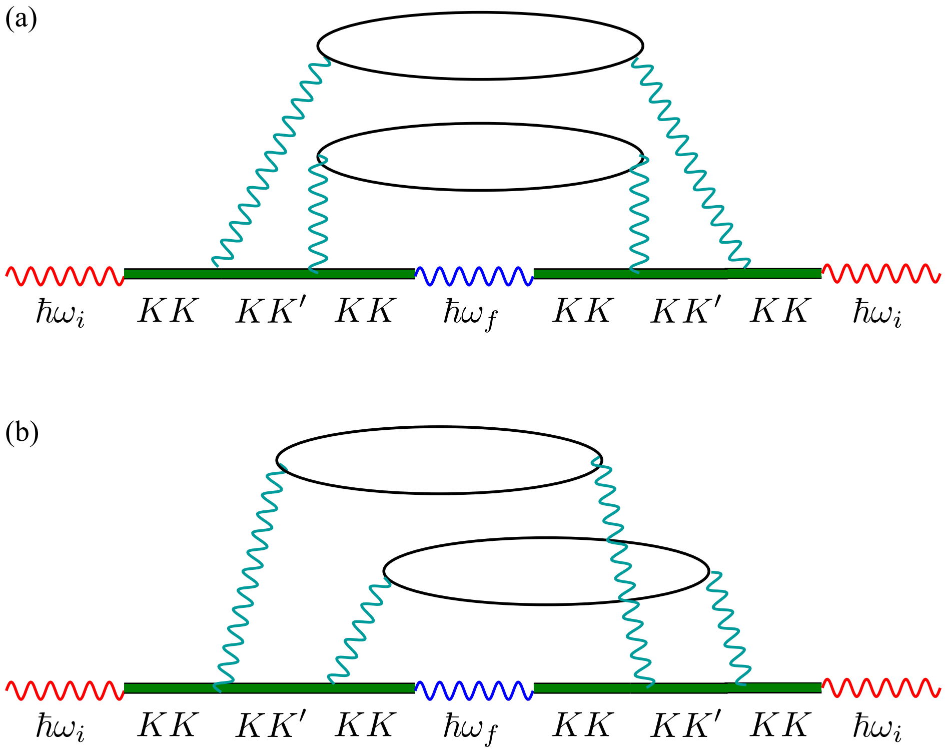

Figure 3: Diagrams responsible for the electron-scattering induced upconversion. KK and KK' denote the intra- and inter-valley excitons in the intermediate and final states, cyan-colored lines describe the Coulomb electron-electron interaction, red and blue wavy lines show the Green's functions for the photons in the initial and final states, respectively, loops represent the electron polarization operators.

In the non-crossing approximation, where only the diagram in Fig. 3(a) is taken into

account, we obtain the expression for the upconversion rate, Fig. 3:[1]

$$S_{up}^{ee} \propto |V_0|^4 \frac{1}{(\hbar\omega_f - E_X)^2 + \hbar^2\gamma^2} \frac{1}{(\hbar\omega_{exc} - E_X)^2 + \hbar^2\gamma^2} \times \sum_{\boldsymbol{q}} \int \frac{d\omega}{2\pi} \Pi(\omega, \boldsymbol{q}) \Pi(\omega_f - \omega_{exc} - \omega, \boldsymbol{q}) |G_{IX}(\hbar\omega_{exc} - E_{IX} + \hbar\omega, \boldsymbol{q})|^2, \quad (5)$$

where we neglected the small wavevectors of the incident and emitted photons as compared to the electron wavevector $\boldsymbol{q} = \boldsymbol{k} - \boldsymbol{K}$, with the transferred wavevector $\boldsymbol{k}$ and the wavevector $\boldsymbol{K}$ connecting the $\boldsymbol{K}_+$ and $\boldsymbol{K}_-$ valleys, $G(\varepsilon, \boldsymbol{q}) = (\varepsilon - \hbar^2 q^2/2m_{IX} + \mathrm{i}\gamma)^{-1}$ is the Green's function of the indirect exciton, and V_0 is the matrix element of the inter-valley electron-electron scattering. In Eq. (5) $\Pi(\omega, \boldsymbol{q})$ is the imaginary part of the electron polarization loop related to the inter-valley scattering

$$\Pi(\omega, \boldsymbol{q}) = \sum_{\boldsymbol{p}} \tilde{f}_{\boldsymbol{p}} (1 - f_{\boldsymbol{p}+\boldsymbol{q}}) \delta(\hbar\omega - E_{\boldsymbol{p}} - \Delta_c + E_{\boldsymbol{p}+\boldsymbol{q}}), \quad (6)$$

with $E_{\boldsymbol{p}} = \hbar^2 p^2/2m_e$ is the electron (e) dispersion, $\tilde{f}_{\boldsymbol{p}}$ and $f_{\boldsymbol{p}+\boldsymbol{q}}$ are the electron distribution functions in the upper and lower spin subbands of the conduction band. To obtain the analytical result we assume that

$$k_B T, E_F \ll \Delta_c. \quad (7)$$

It enables us to neglect the occupancy of the final states and also to replace $\boldsymbol{p} + \boldsymbol{q}$ by $\boldsymbol{q}$ since $p \sim \sqrt{2m_e k_B T/\hbar^2} \ll q \lesssim \sqrt{2m_e \Delta_c/\hbar^2}$. Consequently,

$$\Pi(\omega, \boldsymbol{q}) = \tilde{N}_e \delta(\hbar\omega - \Delta_c + E_{\boldsymbol{q}}), \quad (8)$$

where $\tilde{N}_e$ is the electron occupancy in the two-spin subband. Hence, it follows from Eq. (5)

[1] The diagram with crossing of the Coulomb lines, Fig. 2(b) is typically small, cf. discussion in Ref. [10].

that

$$S_{up}^{ee} \propto |V_0|^4 \frac{1}{(\hbar\omega_f - E_X)^2 + \hbar^2\gamma^2} \frac{1}{(\hbar\omega_{exc} - E_X)^2 + \hbar^2\gamma^2}$$
$$\times \frac{\tilde{N}_e^2}{2\pi} \sum_{\boldsymbol{q}} \frac{\delta(\hbar\omega_f - \hbar\omega_{exc} - 2\Delta_c + 2\frac{\hbar^2 q^2}{2m_e})}{\gamma^2 + \left[\hbar\omega_{exc} - E_{IX} + \Delta_c - \frac{\hbar^2 q^2}{2m_e}\left(1 + \frac{m_e}{m_{IX}}\right)\right]^2}$$
$$= |V_0|^4 \frac{1}{(\hbar\omega_f - E_X)^2 + \hbar^2\gamma^2} \frac{1}{(\hbar\omega_{exc} - E_X)^2 + \hbar^2\gamma^2}$$
$$\times \frac{\tilde{N}_e^2}{2\pi} \mathcal{D}_e \frac{\Theta(2\Delta_c - \hbar\omega_f + \hbar\omega_{exc})}{\hbar^2\gamma^2 + \delta^2}, \quad (9)$$

where

$$\delta = [(\hbar\omega_f - \hbar\omega_{exc}) - 2\Delta_c] \frac{m_e}{2m_{IX}} + \frac{\hbar\omega_{exc} + \hbar\omega_f}{2} - E_{IX}. \quad (10)$$

Taking into account that the upconverted PL is observed at the exciton resonance $\hbar\omega_f \approx E_X$, and introducing the notations

$$\Delta = E_X - E_{IX} \quad \text{(the splitting between the direct and indirect excitons)}, \quad (11)$$

$$\sigma = m_e/m_{IX} \quad \text{(the electron to exciton mass ratio)}, \quad (12)$$

$$\tilde{\gamma}_{ee} = \tilde{N}_e \mathcal{D}_e |V_0|^2 \quad \text{(the characteristic e-e scattering rate)}, \quad (13)$$

we recast the upconversion rate in the form similar to Eq. (2):

$$S_{up}^{ee} \propto \frac{\tilde{N}_e \gamma}{\hbar^2\gamma^2 + \left(\frac{1-\sigma}{2}\right)^2 (\hbar\omega_{exc} - E_*)^2} \frac{1}{(\hbar\omega_f - E_X)^2 + \hbar^2\gamma^2} \frac{1}{(\hbar\omega_f - \hbar\omega_{exc})^2} \frac{\tilde{\gamma}_{ee}}{\gamma}. \quad (14)$$

Hence, the resonance in the upconversion excitation is at

$$E_* = E_X - \frac{2}{1-\sigma}(\Delta - \Delta_c \sigma). \quad (15)$$

It is worthwhile to note that the broadening of the resonance is underestimated (it is in the order of $k_B T$ due to the spread of the electron energies in the upper subband). We indicate

that Eq. (15) is valid provided that

$$\Delta_c \geqslant \Delta \quad \text{at} \quad 0 < \sigma < 1,$$

otherwise there is no real intermediate state, see discussion below.

In order to illustrate the position of peak we present an elementary derivation of Eq. (15). At the first stage of the process the energy conservation law reads

$$\hbar\omega_{exc} + \Delta_c - \frac{\hbar^2 q^2}{2m_e} = E_{IX} + \frac{\hbar^2 q^2}{2m_{IX}}. \tag{16a}$$

At the second stage, the energy conservation reads

$$E_{IX} + \frac{\hbar^2 q^2}{2m_{IX}} + \Delta_c - \frac{\hbar^2 q^2}{2m_e} = E_X. \tag{16b}$$

Expressing q^2 from Eq. (16b) gives us

$$(\sigma - 1)\frac{\hbar^2}{2m_e}q^2 = E_X - E_{IX} - \Delta_c = \Delta - \Delta_c.$$

In this expression $q^2 > 0$ (intermediate state is real); thus, for $\sigma < 1$ (exciton is heavier than the electron) we should have $\Delta_c > \Delta$ to obtain a resonant electron-assisted process. Substituting this expression into Eq. (16a) we have

$$\hbar\omega_{exc} = E_X - \Delta + (\sigma + 1)\frac{\hbar^2}{2m_e}q^2 - \Delta_c = E_X - \Delta_c - \Delta + \frac{\sigma + 1}{\sigma - 1}(\Delta - \Delta_c),$$

which reduces to Eq. (15).

Rough estimation: For $\Delta = \Delta_c$ and $\sigma = 1/2$, we obtain

$$E_* \approx E_X - 2\Delta = E_{IX} - \Delta = E_{IX} - \Delta_c. \tag{17}$$

The general case of $\Delta > \Delta_c$ is less trivial, since there is no resonance in the intermediate state. The analysis of the general Eq. (5) in the limit of $T \to 0$ and $\gamma \to 0$ shows that, for $0 < \sigma < 1$, two steps are expected:

$$E_{*,1} = E_X - 2\Delta_c, \quad E_{*,2} = E_X - \Delta - \Delta_c. \tag{18}$$

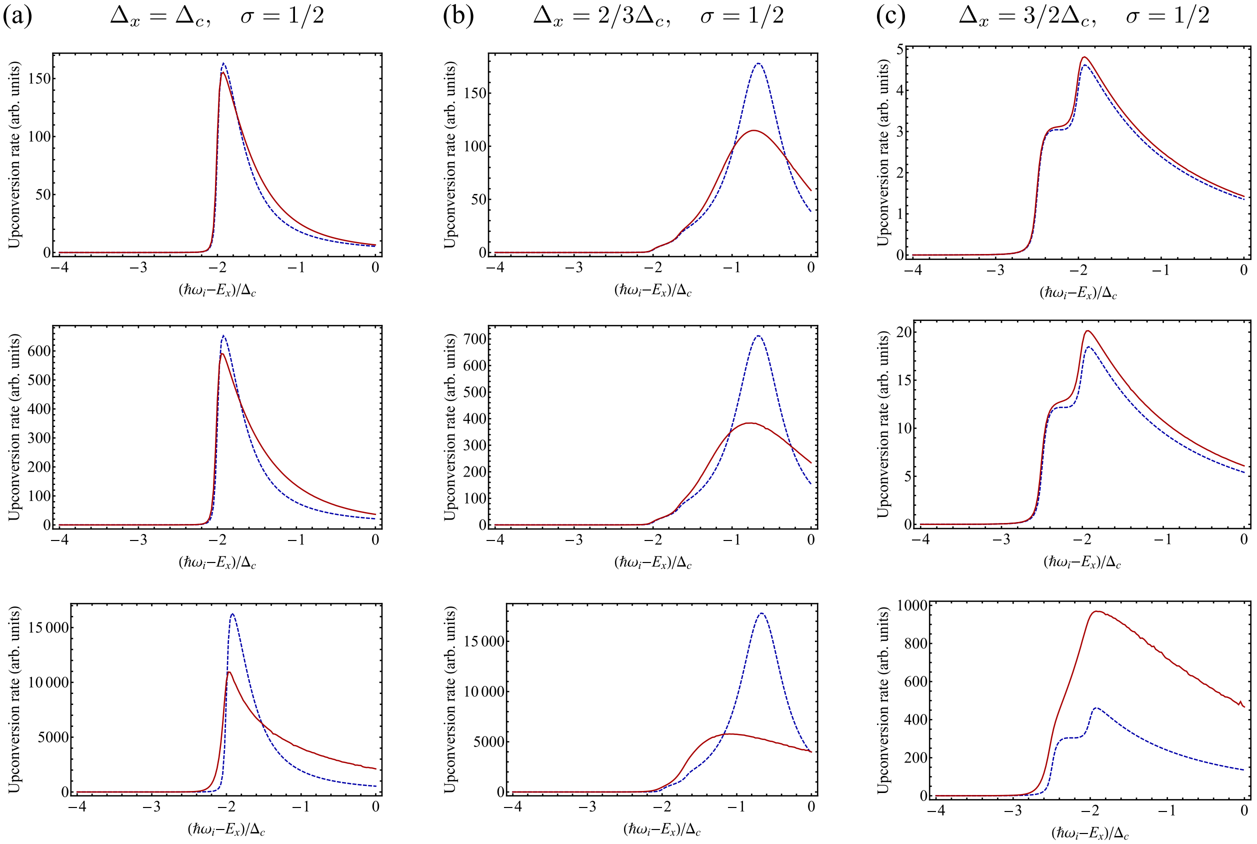

Figure 4: Upconversion rates as a function of detuning, for different parameters. Column (a) $\Delta = \Delta_c$, $\sigma = 1/2$; column (b) $\Delta = (2/3)\Delta_c$, $\sigma = 1/2$, and column (c) $\Delta = (3/2)\Delta_c$, $\sigma = 1/2$. Top, middle, and bottom rows correspond to $k_B T = \Delta_c/100$, $\Delta_c/50$, and $\Delta_c/10$, respectively. Solid red curves show the results of the full calculation after Eqs. (5) and (6) (neglecting, however, populations of the final electron states), dashed blue curves correspond to the simplified model with Π given by Eq. (8). All resonances are artificially broadened by $\gamma = \Delta_c/5$.

Figure 4 shows the upconversion rates calculated numerically using the full expression of the electron polarization operator, for four cases of interest:

(a) $\Delta = \Delta_c$, $\sigma = 1/2$ with the peak at $\hbar\omega = E_X - 2\Delta$ in accordance with Eq. (15).

(b) $\Delta = (2/3)\Delta_c$, $\sigma = 1/2$ with the peak at $\hbar\omega = E_X - \Delta$ in accordance with Eq. (15).

(c) $\Delta = (3/2)\Delta_c$, $\sigma = 1/2$ with the steps at $\hbar\omega_{exc} = E_X - \Delta - \Delta_c$ and $\hbar\omega_{exc} = E_X - 2\Delta_c$ in accordance with Eq. (18).

As compared to the analytical results, the smearing of the peaks with increasing temperature is clearly seen.

Electron & defect processes

Let us consider another type of process, in which the energy is taken from the electron, while the momentum can be dissipated by a defect. In this case there are two processes: (i) After the virtual absorption of a photon, the exciton scatters to the opposite valley by a defect and then, in a next step, by an electron back to the initial valley, and (ii) first scattering is performed by an electron and the second one is done by the defect.

Let us analyze the energy and momentum conservation laws in more detail considering, *e.g.*, the pathway (i):

$$\hbar\omega_{exc} = E_{IX} + \frac{\hbar^2 q^2}{2m_{IX}}, \tag{19a}$$

$$\hbar\omega_f = E_X = \hbar\omega_{exc} + \Delta_c + \frac{\hbar^2 p^2}{2m_e} - \frac{\hbar^2(\boldsymbol{q}+\boldsymbol{p})^2}{2m_e}. \tag{19b}$$

Our goal is to find a threshold for the resonant upconversion process. Combining Eqs. (19), we obtain

$$\frac{\hbar^2 q^2}{2m_e}(1-\sigma) + \frac{\hbar^2}{m_e} pq\cos\vartheta + \Delta - \Delta_c = 0. \tag{20}$$

Here, p and q are the absolute values of the wavevectors and ϑ is the angle between p and q. Equation (20) has a real solution for q at

$$p^2\cos^2\vartheta > \frac{2m_e}{\hbar^2}(1-\sigma)(\Delta - \Delta_c). \tag{21}$$

In this case

$$q = q_* = -\frac{p\cos\vartheta}{1-\sigma}, \tag{22}$$

which requires the solution with $\cos\vartheta < 0$ and, for the threshold energy, we have

$$\hbar\omega_{exc} > E_* = E_X - \Delta + (\Delta - \Delta_c)\frac{\sigma}{1-\sigma} = E_X - \frac{1-2\sigma}{1-\sigma}\Delta - \frac{\sigma}{1-\sigma}\Delta_c. \tag{23}$$

Interestingly, for $\sigma = 1/2$ (the exciton is twice heavier than the electron), the threshold is at $E_* = E_X - \Delta_c$.

Upconversion through the trion (Fermi-polaron)

The mechanisms of the phonon-assisted PL upconversion *via* the trion have been studied in Ref.[13]. Also, the electron-electron scattering can result in the upconversion as follows:

$$X^- + e' \to X + 2e,$$

where the prime denotes the free resident electron in the excited subband. Here, at the resonance, $\hbar\omega_{exc} = E_T$ (the trion energy) and $\hbar\omega_f = E_X$ (the exciton energy).

To conclude this section we also note that the upconversion can take place *via* further processes: For example, in the course of phonon- or electron-assisted upconversion, a direct exciton with non-zero momentum can be formed and can relax towards to the radiative state. The detailed analysis of such processes is beyond the scope of the present work.

Helicity-resolved Raman scattering spectra

Figures 5(a) and 5(b) present low-temperature (7 K) helicity-resolved Raman scattering spectra of the hBN-encapsulated WSe_2 monolayer excited nearly resonantly at the neutral exciton energy with 707 nm (1.753 eV) and 704.5 nm (1.760 eV), respectively. The exciting

laser light is σ^+ polarized and the detection is either σ^+ or σ^- polarized. The polarization configurations are labeled by $\sigma^+ \, \sigma^+$ (red line) or $\sigma^+ \, \sigma^-$ (blue line).

The Raman spectra identify first-order, energetically degenerated A_1' and E' optical phonons with energies 31 meV (250 cm^{-1})[14]. The out-of-plane A_1' mode is visible only in the $\sigma^+ \, \sigma^+$ configuration, whereas the in-plane E' is detected only in the spectra of opposite helicity $\sigma^+ \, \sigma^-$[15].

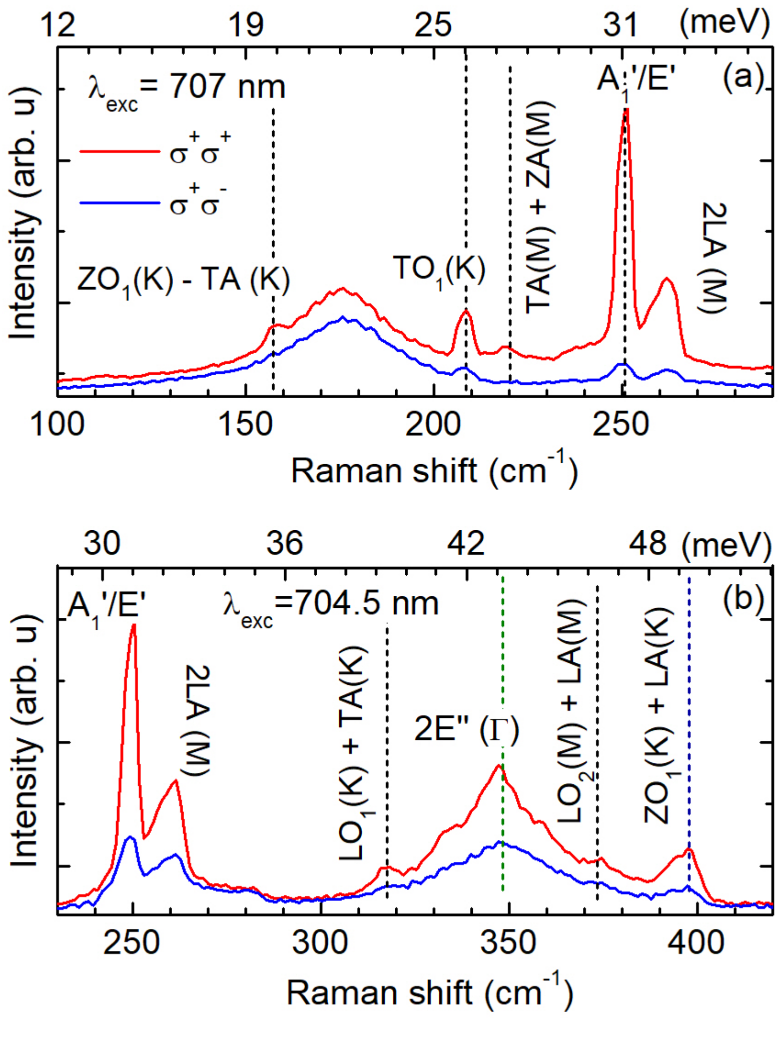

Figure 5: Helicity-resolved Raman scattering spectra excited resonantly at (a) 707 nm and (b) 704.5 nm.

The second-order processes are also visible in the Raman spectra and reveal different polarization properties. In Fig. 5(a) the Raman scattering peak at 158.3 cm^{-1} (20 meV) is assigned to the combination of phonon modes from the K-point in the Brillouin zone (BZ)[14,16];

ZO_1(K)-TA(K) or LO_2(K)-TA(K), whereas peaks at 219.3 cm^{-1} (27 meV) and 261 cm^{-1} are attributed to the combination of phonon modes from the M-point[14,16]; TA(M)+ZA(M) and 2LA(M), correspondingly. The Raman features at 158.3 cm^{-1} and at 219.3 cm^{-1} are well resolved in the σ^+ σ^+ configuration. The peak at 208.7 cm^{-1} (26 meV) is assigned to the TO_1(K) mode[14] (or TO(E")) branch at the K-point[17]) and is observed in both configurations; however, it is much more intense in the σ^+ σ^+ configuration.

Evaluating the Raman spectra in Fig. 5(b), we can distinguish additional Raman bands. The mode at 318 cm^{-1} (40 meV) is allocated to the combination of phonon modes from the K-point[14]: LO_1(K)+TA(K).

Polarization-resolved upconversion PL spectra

Figures 6(a), (b) and (c) show polarization-resolved UPC PL spectra measured at 40 K for different energy gains of 20 meV, 28 meV, and 34 meV, respectively. The first energy gain is comparable to the binding energy of the XX^0 and resonates with the energy of the E'' phonon. The second one is related to the phonon-mediated upconversion from the inter-valley trion T_T to the exciton X. The third one is assigned to the UPC process from the inter-valley momentum-dark exciton (I_1) to the neutral exciton X. The energy scale of each UPC PL spectrum is shown with respect to the neutral exciton energy ($E - E_X$). All spectra are excited with σ^+ circularly polarized light and are detected with the same (σ^+, red line) or opposite helicity (σ^-, blue line). As seen in Fig. 6(a) the upconversion PL of the X is co-polarized to the laser excitation with $P_{deg} = 43\%$. It is also consistent with the polarization properties of the E'' phonon which couples bright and dark excitons in the K-valley; see Fig.5(b).

Figure 6(b) presents polarization-resolved UPC PL spectra at 40 K for a higher energy gain, excited nearly resonantly at the T_T state. The UPC PL of both the X and XX^0 peaks is co-polarized to the laser light with $P_{deg} = 33\%$ and $P_{deg} = 57\%$, respectively. The circular

polarization degree of the upconverted X emission is lower than that for the excitation in resonance with the XX^0 state. It is comparable with values obtained in regular PL experiments under nearly resonant excitation[18]. It is likely caused by the fact that bright excitons may be scattered into the momentum-dark exciton states due to the interaction with K-point phonons or electrons. It is also worth to mention that the preservation of the exciton valley polarization, for excitation at the T_T energy, is consistent with the polarization properties of the A_1' phonon[19].

Figure 6(c) shows polarization-resolved UPC PL spectra excited nearly resonantly at the I_1 energy. The upconverted emission of the X and XX^0 peaks is also co-polarized to the exciting light with $P_{\text{deg}} = 29\%$ and $P_{\text{deg}} = 56\%$, respectively. Both the values are comparable with values obtained in regular PL experiments under nearly resonant excitation[18]. On the whole, the upconversion PL is co-polarized with the excitation which supports our model, where hole inter-valley transitions are disregarded.

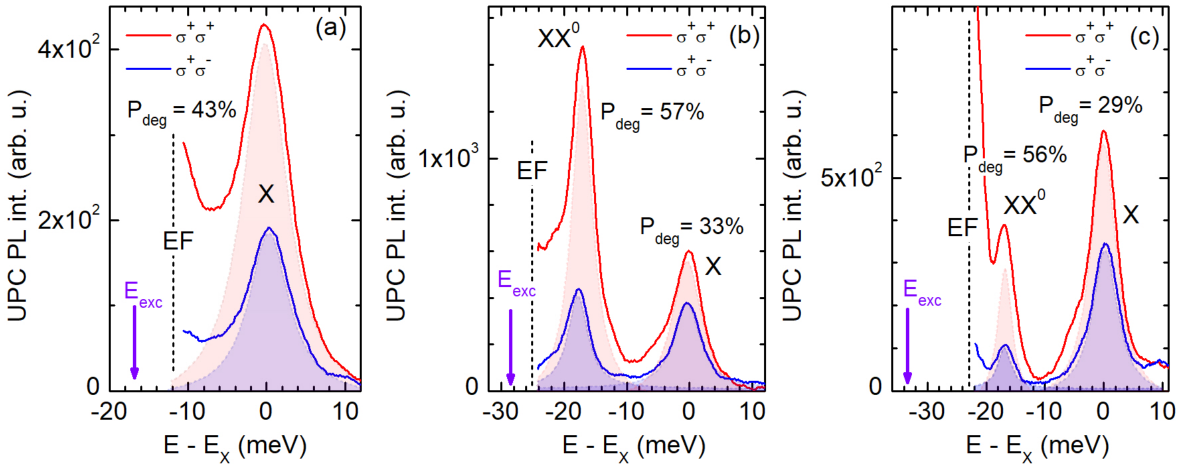

Figure 6: Polarization-resolved UPC PL spectra measured at 40 K, for sample f_2 and different excitation energies E_{exc}: (a) 20 meV below the X, (b) 28 meV below the X and (c) 34 meV below the X. In each panel the spectral cut-off of the edge filter (EF) is indicated by a dashed line.

References

1. Ovsyankin, V.; Feofilov, P. Mechanism of Summation of Electronic Excitations in Activated Crystals. *Soviet Journal of Experimental and Theoretical Physics Letters* **1966**, *3*, 322.

2. Seidel, W.; Titkov, A.; André, J. P.; Voisin, P.; Voos, M. High-Efficiency Energy Up-Conversion by an "Auger Fountain" at an InP-AlInas Type-II Heterojunction. *Phys. Rev. Lett.* **1994**, *73*, 2356–2359.

3. Chen, W.; Joly, A. G.; McCready, D. E. Upconversion Luminescence from CdSe Nanoparticles. *The Journal of Chemical Physics* **2005**, *122*.

4. Eshlaghi, S.; Worthoff, W.; Wieck, A. D.; Suter, D. Luminescence Upconversion in GaAs Quantum Wells. *Phys. Rev. B* **2008**, *77*, 245317.

5. Chen, S. L.; Stehr, J.; Reddy, N. K.; Tu, C. W.; Chen, W. M.; Buyanova, I. A. Efficient Upconversion of Photoluminescence *via* Two-Photon Absorption in Bulk and Nanorod ZnO. *Applied Physics B* **2012**, *108*, 919–924.

6. Jones, A. M.; Yu, H.; Schaibley, J. R.; Yan, J.; Mandrus, D. G.; Taniguchi, T.; Watanabe, K.; Dery, H.; Yao, W.; Xu, X. Excitonic Luminescence Upconversion in a Two-Dimensional Semiconductor. *Nat Phys* **2016**, *12*, 323–327.

7. Manca, M.; Glazov, M. M.; Robert, C.; Cadiz, F.; Taniguchi, T.; Watanabe, K.; Courtade, E.; Amand, T.; Renucci, P.; Marie, X.; Wang, G.; Urbaszek, B. Enabling Valley Selective Exciton Scattering in Monolayer WSe_2 through Upconversion. *Nature Communications* **2017**, *8*, 14927.

8. Han, B.; Robert, C.; Courtade, E.; Manca, M.; Shree, S.; Amand, T.; Renucci, P.; Taniguchi, T.; Watanabe, K.; Marie, X.; Golub, L. E.; Glazov, M. M.; Urbaszek, B.

Exciton States in Monolayer $MoSe_2$ and $MoTe_2$ Probed by Upconversion Spectroscopy. *Phys. Rev. X* **2018**, *8*, 031073.

9. Jadczak, J.; Bryja, L.; Kutrowska-Girzycka, J.; Kapuściński, P.; Bieniek, M.; Huang, Y. S.; Hawrylak, P. Room Temperature Multi-Phonon Upconversion Photoluminescence in Monolayer Semiconductor WS_2. *Nature Communications* **2019**, *10*, 107.

10. Paradisanos, I.; Wang, G.; Alexeev, E. M.; Cadore, A. R.; Marie, X.; Ferrari, A. C.; Glazov, M. M.; Urbaszek, B. Efficient Phonon Cascades in WSe_2 Monolayers. *Nature Communications* **2021**, *12*, 538.

11. Dery, H. Theory of Intervalley Coulomb Interactions in Monolayer Transition-Metal Dichalcogenides. *Phys. Rev. B* **2016**, *94*, 075421.

12. Van Tuan, D.; Scharf, B.; Žutić, I.; Dery, H. Marrying Excitons and Plasmons in Monolayer Transition-Metal Dichalcogenides. *Phys. Rev. X* **2017**, *7*, 041040.

13. Ayari, S.; Jaziri, S.; Ferreira, R.; Bastard, G. Phonon-Assisted Exciton/Trion Conversion Efficiency in Transition Metal Dichalcogenides. *Phys. Rev. B* **2020**, *102*, 125410.

14. Luca, M. D.; Cartoixà, X.; Martín-Sánchez, J.; López-Suárez, M.; Trotta, R.; Rurali, R.; Zardo, I. New Insights in the Lattice Dynamics of Monolayers, Bilayers, and Trilayers of WSe_2 and Unambiguous Determination of Few-Layer-Flakes' Thickness. *2D Materials* **2020**, *7*, 025004.

15. Chen, S.-Y.; Zheng, C.; Fuhrer, M. S.; Yan, J. Helicity-Resolved Raman Scattering of MoS_2, $MoSe_2$, WS_2, and WSe_2 Atomic Layers. *Nano Letters* **2015**, *15*, 2526–2532.

16. Norouzzadeh, P.; Singh, D. J. Thermal Conductivity of Single-Layer WSe_2 by a Stillinger–Weber Potential. *Nanotechnology* **2017**, *28*, 075708.

17. Zhu, H.; Yi, J.; Li, M.-Y.; Xiao, J.; Zhang, L.; Yang, C.-W.; Kaindl, R. A.; Li, L.-J.; Wang, Y.; Zhang, X. Observation of Chiral Phonons. *Science* **2018**, *359*, 579–582.

18. Jadczak, J.; Kutrowska-Girzycka, J.; Schindler, J. J.; Debus, J.; Watanabe, K.; Taniguchi, T.; Ho, C.-H.; Bryja, L. Investigations of Electron-Electron and Interlayer Electron-Phonon Coupling in van der Waals hBN/WSe_2/hBN Heterostructures by Photoluminescence Excitation Experiments. *Materials* **2021**, *14*, 399.

19. Jones, A. M.; Yu, H.; Schaibley, J. R.; Yan, J.; Mandrus, D. G.; Taniguchi, T.; Watanabe, K.; Dery, H.; Yao, W.; Xu, X. Excitonic Luminescence Upconversion in a Two-Dimensional Semiconductor. *Nature Physics* **2016**, *12*, 323–327.
